# Clinical and Pathological Heterogeneity of Cervical Intraepithelial Neoplasia Grade 3

**DOI:** 10.1371/journal.pone.0029051

**Published:** 2012-01-13

**Authors:** Hannah P. Yang, Rosemary E. Zuna, Mark Schiffman, Joan L. Walker, Mark E. Sherman, Lisa M. Landrum, Katherine Moxley, Michael A. Gold, S. Terence Dunn, Richard A. Allen, Roy Zhang, Rodney Long, Sophia S. Wang, Nicolas Wentzensen

**Affiliations:** 1 Division of Cancer Epidemiology and Genetics, National Cancer Institute, Rockville, Maryland, United States of America; 2 Department of Pathology, University of Oklahoma Health Sciences Center, Oklahoma City, Oklahoma, United States of America; 3 Department of Obstetrics and Gynecology, University of Oklahoma Health Sciences Center, Oklahoma City, Oklahoma, United States of America; 4 Department of Obstetrics and Gynecology, Vanderbilt University, Nashville, Tennessee, United States of America; 5 National Library of Medicine, Bethesda, Maryland, United States of America; 6 Division of Cancer Etiology, Beckman Research Institute, City of Hope, Duarte, California, United States of America; Vanderbilt University, United States of America

## Abstract

**Objective:**

Cervical intraepithelial neoplasia grade 3 (CIN3), the immediate cervical cancer precursor, is a target of cervical cancer prevention. However, less than half of CIN3s will progress to cancer. Routine treatment of all CIN3s and the majority of CIN2s may lead to overtreatment of many lesions that would not progress. To improve our understanding of CIN3 natural history, we performed a detailed characterization of CIN3 heterogeneity in a large referral population in the US.

**Methods:**

We examined 309 CIN3 cases in the SUCCEED, a large population-based study of women with abnormal cervical cancer screening results. Histology information for 12 individual loop electrosurgical excision procedure (LEEP) segments was evaluated for each woman. We performed case-case comparisons of CIN3s to analyze determinants of heterogeneity and screening test performance.

**Results:**

CIN3 cases varied substantially by size (1–10 LEEP segments) and by presentation with concomitant CIN2 and CIN1. All grades of CINs were equally distributed over the cervical surface. In half of the women, CIN3 lesions were found as multiple distinct lesions on the cervix. Women with large and solitary CIN3 lesions were more likely to be older, have longer sexual activity span, and have fewer multiple high risk HPV infections. Screening frequency, but not HPV16 positivity, was an important predictor of CIN3 size. Large CIN3 lesions were also characterized by high-grade clinical test results.

**Conclusions:**

We demonstrate substantial heterogeneity in clinical and pathological presentation of CIN3 in a US population. Time since sexual debut and participation in screening were predictors of CIN3 size. We did not observe a preferential site of CIN3 on the cervical surface that could serve as a target for cervical biopsy. Cervical cancer screening procedures were more likely to detect larger CIN3s, suggesting that CIN3s detected by multiple independent diagnostic tests may represent cases with increased risk of invasion.

## Introduction

The natural history of human papillomavirus (HPV) leading to invasive cervical cancer is well established [1]. Genital HPV infections are very common in sexually active women, but most infections regress spontaneously. Few infections persist and progress to pre-cancer, diagnosed histologically as cervical intraepithelial neoplasia grade 3 (CIN3) [2]. Primary prevention by HPV vaccination or secondary prevention by screening for and removing a cancer precursor before invasion occurs are currently the basis for cervical cancer prevention [2]. A confirmed CIN3 is typically treated by the loop electrosurgical excision procedure (LEEP) according to American Society for Colposcopy and Cervical Pathology (ASCCP) guidelines [3]. However, only 30–50% of advanced CIN3 lesions progress to cancer [4], [5], indicating that there is a variability of risk of invasion to cervical cancer within a group collectively defined as CIN3 [6]. Overtreatment with LEEP may put women at unnecessary risk of side effects such as bleeding and infection [7] as well as potential negative impact on reproductive outcomes for young women [8].

Reporting CIN3 as a single outcome based on the worst histological diagnosis on the cervix [5], [9], [10], [11] does not capture the complexity of the histological patterns on the cervical surface or reflect the heterogeneous risk associated with CIN3. Currently, the heterogeneity of CIN3 is not well understood and there are no certain phenotypic features of CIN3 that predict risk of progression, except possibly for HPV genotype, that could be used for clinical management [2]. CIN3 lesion size, that is extension of CIN3s around the cervical epithelial surface, is hypothesized to be associated with risk of progression [5], [10].

Detailed mapping of the LEEP segments that covers the surface of the entire cervix provides an opportunity to identify and characterize clinical subgroups of CIN3 cases. Thus, we performed a detailed characterization of LEEP specimens to understand predictors of CIN3 heterogeneity and to evaluate the relationship of CIN3 heterogeneity with screening test results in a large population-based study of women with abnormal cervical cancer screening results. Examining the heterogeneity CIN3 cases referred to a colposcopy clinic after abnormal cervical cancer screening may aid in elucidating the biological differences between CIN3 cases, and thereby inform future efforts to reduce unnecessary treatment of CIN3 that are not clinically important.

## Materials and Methods

### Study Population

We conducted the analysis in the Study to Understand Cervical Cancer Early Endpoints and Determinants (SUCCEED), a large population-based study composed of women referred to the University of Oklahoma Health Sciences Center (OUHSC) for abnormal cervical cancer screening test results. SUCCEED design and methodology, including the details on enrollment, questionnaire data, HPV DNA genotyping, histology, and cytology procedures, have been described in depth elsewhere [12], [13]. In brief, the main component of SUCCEED was conducted between 2003 and 2007 by inviting women referred to colposcopy at the OUHSC Dysplasia Clinic following an abnormal Pap smear result or a biopsy diagnosis of CIN. Continued accrual of women specifically with CIN3 and cancers lasted until March 2010. Written informed consent was obtained from all women enrolled into the study and Institutional Review Board approval was provided by OUHSC and the US National Cancer Institute.

### Questionnaire and Colposcopy

Participants completed interviewer-administered, standardized questionnaires and provided liquid-based cytology specimens for ThinPrep Pap and HPV genotyping by Linear Array (Roche Diagnostics). OUHSC gynecologists performed colposcopic examination according to routine OUHSC practice. Women were treated by LEEP of the transformation zone, if indicated by ASCCP guidelines [3].

### LEEP Histopathology

Every LEEP specimen was divided into 12 topographically designated sections or segments (a “clockface” depiction of the cervix) for detailed histopathological mapping. According to the SUCCEED study protocol, two segments from each LEEP representing the worst lesion and normal cervical tissue were snap-frozen for molecular studies. The remaining 10 segments were formalin-fixed, paraffin-embedded and analyzed to generate individual histology results for each segment. The study pathologists at OUHSC, masked to HPV genotyping data, determined the histology using CIN terminology. One or more of the following diagnoses were noted for each o'clock segment of the cervix per individual: other, negative/normal, atypical metaplasia, CIN1, CIN2, CIN3, adenocarcinoma in situ, squamous cell carcinoma, and adenocarcinoma. In addition, if the clinician determined that the entire transformation zone or extent of a lesion could not be visualized adequately, endocervical curettage (ECC) and/or a deeper, secondary LEEP (“top hat procedure”), which removes tissue from higher up in the endocervical canal, were performed. Per common practice, the cases were categorized according to the worst diagnosis for each woman based on the diagnosis of the most abnormal LEEP segment, ECC, and/or top hat.

### Analytic Population

**Figure 1:**
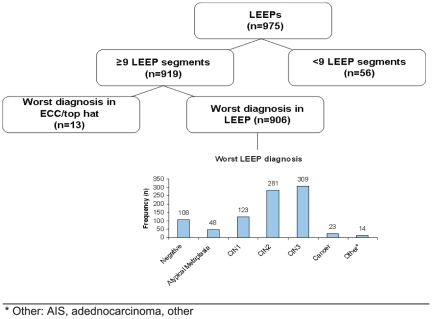
LEEPs performed among SUCCEED women in a colposcopy clinic, 2003–2010.

During the study period, 975 women were managed by LEEP (**Figure 1**). We excluded 56 women that had more than three of 12 missing LEEP segments, which precluded a total assessment of the number of various subgroups of CIN3 and size of CIN3. Among the remaining 919 women (94% of n = 975), the worst lesion was found on ECC and/or the top hat procedure in 13 women and on the LEEP in 906 women (93% of n = 975). In all, 353 women were diagnosed with CIN3 in one or more histologic section (either based on results from biopsy or LEEP). For this study, the analytic population was based on the subset of 309 CIN3 cases as defined by the worst diagnosis in the LEEP.

### CIN Endpoints

**Figure 2:**
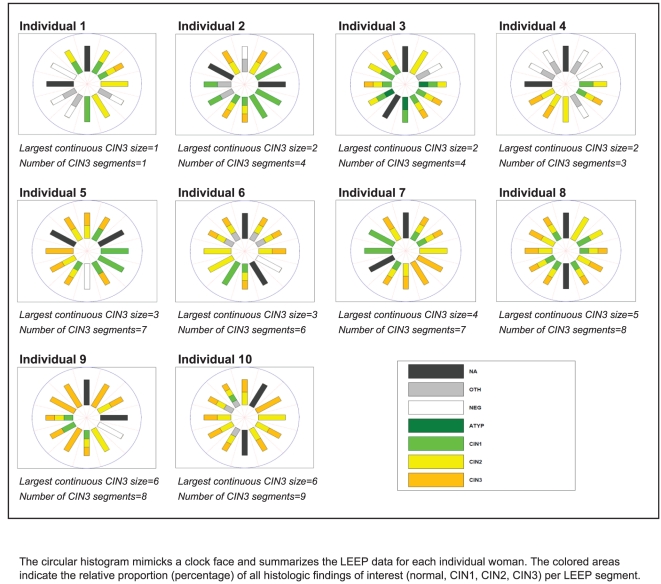
Sample of individual data for depicting the distribution of CIN LEEP segments among women in SUCCEED diagnosed as with CIN3 by LEEP.

**Figure 2** displays a circular histogram of the LEEP diagnoses from a representative sample of ten women. Each circular histogram resembles a clock face and summarizes the LEEP data for each individual woman. The colored areas indicate the histologic findings of interest (normal, CIN1, CIN2, CIN3) per LEEP segment. These randomly selected women represent a spectrum of CIN3 cases that differ in CIN3 lesion size, whether by number of LEEP segments with CIN3 among all LEEP segments analyzed or by number of continuous adjacent LEEP segments with CIN3. These examples highlight the complexity of the diagnoses with the considerable variation in the size of the lesions and presence of different grades of CIN within the same LEEP segment.

For our analysis, we defined the diagnosis of each o'clock segment of the cervix by the most severe diagnosis, if more than one diagnosis was noted for an individual LEEP segment. We focused our analysis on the following worst outcomes for each LEEP segment: normal, CIN1, CIN2, and CIN3. A priori, we defined subgroups of CIN3 cases by the presence or absence of CIN1 and CIN2 in conjunction with the presence of CIN3 in any of the LEEP segments. Accordingly, we categorized the CIN3 cases into four subgroups: solitary CIN3, CIN3+CIN2, CIN3+CIN2+CIN1, and CIN3+CIN1. As a sensitivity analysis, we further categorized solitary CIN3 as “true” solitary CIN3 cases by excluding cases with CIN1 and/or CIN2 diagnoses in any of CIN3 LEEP segments. We also a priori defined the size of CIN3 by the number of LEEP segments with CIN3 among all LEEP segments analyzed and the number of *continuous* adjacent LEEP segments with CIN3. In addition, we dichotomized the CIN3 lesion size as a “small” versus “large” CIN3 lesion, defined by a cut-off of <3 CIN3 segments versus 3+ CIN3 segments. Similar trends were observed with different cut-points (<2 versus 2+, <2 versus 3+, <4 versus 4+, <5 versus 5+).

### Statistical Analyses

First, we performed a case-case comparison of the four CIN3 subgroups for selected known risk factors of cervical cancer: age at LEEP, length span of sexual activity (age difference between ages at sexual initiation and at LEEP), parity, OC use, lifetime number of sexual partners, smoking, Pap test history (number in past five years), HPV genotypes (number of any HPV type infections, number of high risk (HR) HPV type infections, and presence of HPV16 infections). We also performed a case-case comparison for diagnostic factors based on the following cervical cancer screening tests: cytology results prior to LEEP, biopsy results at LEEP visit, and colposcopic impression results at LEEP visit. In addition to the categorical variables of these screening test results, we dichotomized the screening test results to distinguish low grade from high grade diagnoses: biopsy histology of CIN3+ compared with CIN2 or less, cytology of high-grade squamous intraepithelial lesions (HSIL) or worse (HSIL+) compared with less than HSIL, colposcopic impression of CIN3+ compared with CIN2 or less. Second, we evaluated the differences between categories of CIN3 lesion size with the same factors. We tested for differences between categorical variables and CIN3 subgroups and lesion size using the Pearson χ^2^ test. In addition, we tested for trend across ordered groups using the *nptrend* command, a nonparametric test that is an extension of the Wilcoxon rank-sum test. We also calculated the percent detection of small versus large CIN3 lesions by the diagnostic tests. For all analyses, *P*-values of ≤0.05 were considered statistically significant. All tests of statistical significance were two-tailed. Analyses were performed using Stata 11.1 (StataCorp., College Station, TX).

## Results

### Heterogeneous Presentation of CIN3 in a Large Series of LEEPs

Three hundred and nine women were identified as CIN3 cases according to the worst LEEP diagnosis (**Table 1**). The median age was 27 years old (range: 18–76 years old) and median sexual activity span was 11 years (range: 2–59 years). In 155 of 309 (50%) CIN3s, multiple distinct CIN3 lesions were present on the cervix. Overall, we observed a wide range of CIN3 lesion size (i.e. number of segments with a worst diagnosis of CIN3) ranging from 1–10 segments (mean 3.0 segments, standard deviation 2.0 segments). We observed a slightly lower average number of largest *continuous* CIN3 size (data not shown). In addition, we also observed a variable range in number of CIN2 (1–10 segments) and CIN1 (1–6 segments) present as concomitant lesions among the CIN3 cases (data not shown).

**Table 1:** Distribution of age, sexual activity span, and number of CINs among women in SUCCEED diagnosed as CIN3 by LEEP (N = 309).

	All CIN3	
	N = 309	
**Age at LEEP (years)**		
*Median*	27	
<23	57	19%
23–26	80	27%
27–33	91	31%
>33	69	23%
**Sexual activity span (years)**		
*Median*	11	
<7	48	17%
7–11	102	36%
12–17	67	24%
>17	63	23%
**Number of distinct CIN3 clusters (n)**		
*Mean (standard deviation)*	1.6 (0.7)	
1	154	50%
2	121	39%
3	32	10%
4	2	1%
**Number of CIN3 segments (n)**		
*Mean (standard deviation)*	3.0 (2.0)	
1	87	28%
2	70	23%
3	55	18%
4	42	14%
5+	55	18%

**Figure 3:**
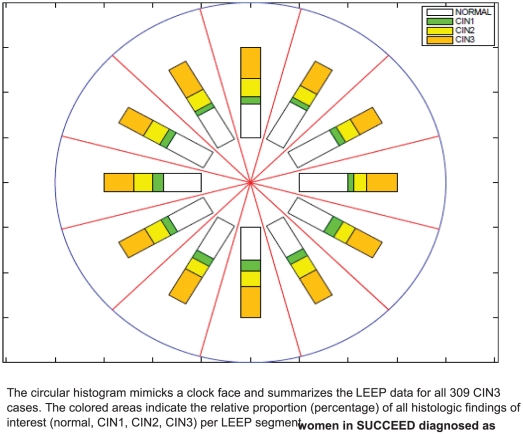
Distribution of CIN LEEP segments among women in SUCCEED diagnosed as CIN3 by LEEP (N = 309).

We summarized all 309 women included in the analysis in a single circular histogram (**Figure 3**). The colored areas indicate the relative proportion (percentage) of all histologic findings of interest (normal, CIN1, CIN2, CIN3) per LEEP segment. All grades of cervical neoplasia were equally distributed over the cervical surface (p>0.05): an average of 43% of each segment had a normal diagnosis (range: 36–50%), 32% had CIN3 (range: 26–36%), 17% had CIN2 (range: 13–20%), and 9% had CIN1 (range: 6–12%).

### Characteristics of CIN3 Subgroups

The majority of these women (n = 230, 74%) had CIN lesions of lower grades in other LEEP segments in addition to the CIN3. About half of the heterogeneous lesions were CIN3 cases with CIN2 lesions (n = 116), followed by CIN3 without additional lesions (n = 79) and CIN3+CIN2+CIN1 (n = 79), as well as a small percentage of CIN3+CIN1 (n = 35) (**Table 2**). Solitary CIN3 and CIN3+CIN1 cases compared to CIN3 cases with CIN2 were more likely to be composed of larger CIN3 lesions. Age at LEEP (p<0.01) and sexual activity span (p<0.01), which were highly correlated (r = 0.97), showed characteristic distributions in CIN3 subgroups. In particular, women presenting with solitary CIN3 were more likely to be older at time of LEEP and have longer sexual activity span compared to other CIN3 subgroups, whereas the CIN3+CIN2+CIN1 subgroup was comprised of the youngest women. Compared to other CIN3 subgroups, solitary CIN3 lesions were more likely to have single HR HPV infections (p = 0.05); there appeared to be a gradation in likelihood of single HR HPV infections from CIN3+CIN1 to CIN3+CIN2+CIN1, to CIN3+CIN2, and to solitary CIN3, although this finding was not statistically significant (**Table 2**). The distributions of other selected risk factors examined, namely number of live births, oral contraceptive use, number of lifetime sexual partners, smoking status, and Pap test frequency, were similar across the subgroups (data not shown).

**Table 2:** Distribution of diagnostic factors among women in SUCCEED diagnosed as CIN3 by LEEP (N = 309).

	CIN3 subgroups								
	Solitary CIN3		CIN3+CIN2		CIN3+CIN2+CIN1		CIN3+CIN1		χ^2^ p-value
	N = 79		N = 116		N = 79		N = 35		
	N	%	N	%	N	%	N	%	
**Number of CIN3 segments (n)**									
*Mean (standard deviation)*	3.6 (2.5)		2.8 (1.9)		2.4 (1.6)		3.5 (2.0)		
1	17	22%	32	28%	31	39%	7	20%	0.02
2	17	22%	28	24%	19	24%	6	17%	
3	12	15%	28	24%	11	14%	4	11%	
4	12	15%	11	9%	10	13%	9	26%	
5+	21	27%	17	15%	8	10%	9	26%	
**Risk Factors**									
**Age at LEEP (years)**									
<23	8	11%	24	22%	22	29%	3	9%	<0.001
23–26	18	24%	25	23%	25	32%	12	34%	
27–33	19	25%	35	32%	21	27%	16	46%	
>33	31	41%	25	23%	9	12%	4	11%	
**Sexual activity span (years)**									
<7	7	10%	21	21%	15	20%	5	14%	0.003
7–11	22	32%	31	30%	35	47%	14	40%	
12–17	13	19%	26	25%	16	22%	12	34%	
>17	27	39%	24	24%	8	11%	4	11%	
**Number of high risk HPV positive** a									
0	7	9%	7	6%	4	5%	1	3%	0.05
1	45	57%	64	55%	30	38%	14	40%	
≥2	27	34%	45	39%	45	57%	20	57%	
**HPV16**									
No	29	37%	36	31%	23	29%	11	31%	0.76
Yes	50	63%	80	69%	56	71%	24	69%	
**Diagnostic Factors**									
**Histology of biopsy prior to LEEP**									
Negative	5	13%	0	0%	1	3%	1	6%	0.09
Atypical Metaplasia	2	5%	1	2%	1	3%	0	0%	
CIN1	2	5%	1	2%	3	8%	2	11%	
CIN2	10	25%	24	38%	12	30%	9	50%	
CIN3	21	53%	38	59%	23	58%	6	33%	
≤CIN2	19	48%	26	41%	17	43%	12	67%	0.25
CIN3+	21	53%	38	59%	23	58%	6	33%	
**Cytology at LEEP**									
Negative	1	1%	2	2%	3	4%	2	6%	0.36
ASC-US, ASC-H, AGUS, LSIL	15	19%	15	13%	18	23%	6	18%	
HSIL, Cancer	62	79%	98	85%	56	73%	26	76%	
<HSIL	16	21%	17	15%	21	27%	8	24%	0.20
HSIL+	62	79%	98	85%	56	73%	26	76%	
**Worst colposcopy impression at LEEP**									
Normal/Equivocal	13	17%	4	4%	1	1%	0	0%	<0.001
CIN1	7	9%	9	8%	6	8%	0	0%	
CIN2	27	36%	55	50%	44	58%	21	60%	
CIN3	26	35%	41	38%	25	33%	14	40%	
Cancer	2	3%	0	0%	0	0%	0	0%	
≤CIN2	47	63%	68	62%	51	67%	21	60%	0.88
CIN3+	28	37%	41	38%	25	33%	14	40%	

a High Risk/Oncogenic HPV type defined as positive for any of the following HPV types: 16, 18, 31, 33, 35, 39, 45, 51, 52, 56, 58, 59, 66, 68.

The distributions of clinical and pathological characteristics examined and presented in **Table 2** were similar across the subgroups (p>0.05).

### Predictors of CIN3 Lesion Size

CIN3 size was widely distributed in the study population and ranged from 87 women (28%) with one CIN3 segment involved to 55 (18%) women with CIN3 across five or more CIN3 segments (**Table 3**). We found borderline significant differences in distribution of age at LEEP (p = 0.06) and sexual activity span (p = 0.07) with CIN3 size; larger CIN3s were found in older women and women with longer sexual activity span (ptrend = 0.02). Importantly, women with larger CIN3 lesions had significantly fewer Pap tests in the past five years (p = 0.04). Of note, HPV16 positivity was not associated with larger CIN3 size (p = 0.73). The distributions of other selected risk factors examined and presented in **Table 3** were similar across CIN3 lesion size (p>0.05).

**Table 3:** Distribution of risk factors and diagnostic factors by CIN3 size among women in SUCCEED diagnosed as CIN3 by LEEP (N = 309).

	Number of CIN3 Segments											
	1		2		3		4		5+		χ^2^ p-value	Trend test p-value
	N = 87		N = 70		N = 55		N = 42		N = 55			
	N	%	N	%	N	%	N	%	N	%		
**Risk Factors**												
**Age at LEEP (years)**												
<23	17	20%	12	17%	13	25%	6	15%	9	17%	0.06	0.02
23–27	25	30%	25	36%	7	14%	14	35%	9	17%		
27–33	30	36%	20	29%	14	27%	10	25%	17	32%		
>33	12	14%	12	17%	17	33%	10	25%	18	34%		
**Sexual activity span (years)**												
<7	14	18%	14	21%	10	21%	4	11%	6	12%	0.07	0.02
7–11	30	38%	31	47%	10	21%	15	39%	16	33%		
12–17	24	30%	10	15%	11	23%	11	29%	11	22%		
>17	12	15%	11	17%	16	34%	8	21%	16	33%		
**Pap test (number in past 5 years)**												
<2	10	15%	10	18%	11	25%	7	22%	19	46%	0.04	0.09
2–3	24	35%	20	36%	10	23%	8	25%	14	34%		
4–5	34	50%	25	45%	23	52%	17	53%	8	20%		
**Number of high risk HPV positive** a												
0	7	8%	3	4%	3	5%	2	5%	4	7%	0.93	0.95
1	45	52%	34	49%	24	44%	21	50%	29	53%		
≥2	35	40%	33	47%	28	51%	19	45%	22	40%		
**HPV16**												
No	26	30%	22	31%	22	40%	13	31%	16	29%	0.73	0.97
Yes	61	70%	48	69%	33	60%	29	69%	39	71%		
**Diagnostic Factors**												
**Histology of biopsy prior to LEEP**												
Negative	4	8%	1	3%	1	3%	1	5%	0	0%	0.29	0.006
Atypical Metaplasia	2	4%	1	3%	0	0%	1	5%	0	0%		
CIN1	3	6%	0	0%	2	6%	2	10%	1	4%		
CIN2	18	37%	17	49%	10	31%	6	30%	4	15%		
CIN3	22	45%	16	46%	19	59%	10	50%	21	81%		
≤CIN2	27	55%	19	54%	13	41%	10	50%	5	19%	0.03	0.006
CIN3+	22	45%	16	46%	19	59%	10	50%	21	81%		
**Cytology at LEEP**												
Negative	2	2%	2	3%	3	6%	1	2%	0	0%	0.02	0.001
ASC-US, ASC-H, AGUS, LSIL	26	30%	11	16%	7	13%	6	14%	4	7%		
HSIL, Cancer	58	67%	56	81%	43	81%	35	83%	50	93%		
<HSIL	28	33%	13	19%	10	19%	7	17%	4	7%	0.01	0.001
HSIL+	58	67%	56	81%	43	81%	35	83%	50	93%		
**Worst colposcopy impression at LEEP**												
Normal+Equivocal	3	4%	5	7%	5	10%	2	5%	3	6%	0.03	0.001
CIN1	9	11%	4	6%	7	14%	2	5%	0	0%		
CIN2	47	57%	41	59%	22	43%	17	43%	20	38%		
CIN3	23	28%	19	28%	16	31%	19	48%	29	56%		
Cancer	1	1%	0	0%	1	2%	0	0%	0	0%		
≤CIN2	59	71%	50	72%	34	67%	21	53%	23	44%	0.01	<0.001
CIN3+	24	29%	19	28%	17	33%	19	48%	29	56%		

a High Risk/Oncogenic HPV type defined as positive for any of the following HPV types: 16, 18, 31, 33, 35, 39, 45, 51, 52, 56, 58, 59, 66, 68.

### Screening Test Results and CIN3 Lesion Size

We examined cytology result, colposcopic impression, and preceding biopsy result in relation to CIN3 size (**Table 3**). In women with larger CIN3 size, a higher percentage of preceding biopsy results was found to be CIN3+ (p = 0.03, p-trend = 0.006). Similarly, larger CIN3 lesions were associated with cytological results of HSIL+ (p = 0.01, p-trend = 0.001), and colposcopic impression of CIN3+ (p = 0.01, p-trend<0.001). Overall, larger CIN3 size was associated with high grade cervical pre-cancer lesion of CIN2+ on preceding biopsy and colposcopic impression, albeit not statistically significant (data not shown). A similar pattern with the screening test results was observed when CIN3 size was defined by the largest *continuous* CIN3 size (data not shown). Dichotomizing CIN3s by lesion size (<3 vs. 3+), the sensitivity of HSIL cytology at LEEP was 74% for small CIN3s and 86% for large CIN3s (**Table 4**). The sensitivity of a CIN3 biopsy prior to LEEP was 45% in small CIN3s and 64% in large CIN3s, and the sensitivity of a CIN3+ colposcopic impression at LEEP was 28% for small lesions and 45% for large lesions.

**Table 4:** Comparison of sensitivity of detecting small versus large CIN3 lesion by diagnostic tests among women in SUCCEED diagnosed as CIN3 by LEEP (N = 309)^a^.

	Small CIN3^b^		Large CIN3^b^	
	n	Sensitivity (%)	n	Sensitivity (%)
**Histology of biopsy prior to LEEP**				
≤CIN2	46	45%	28	64%
CIN3+	38		50	
**Cytology at LEEP**				
<HSIL	41	74%	21	86%
HSIL+	114		128	
**Worst colposcopy impression at LEEP**				
≤CIN2	109	28%	78	45%

a Numbers do not add up to n = 309 because of missing data for diagnostic test results.

b Small CIN3 lesions = <3 CIN3 segments; Large CIN3 lesion = 3+ CIN3 segments.

## Discussion

We characterized LEEP specimens of 309 CIN3 cases from a US referral population. The majority of CIN3 cases (74%) presented with CIN3 lesions in conjunction with lower grades of CIN in different regions on the cervix. We observed a wide range of CIN3 sizes from small focal lesions to extensive CIN3 covering most of the cervix. To better characterize women within the CIN3 diagnostic group, we examined the distribution of risk factors as well as clinical and pathological information from cervical cancer screening tests by CIN3 subgroups and CIN3 lesion size.

Some previous studies have suggested that CIN may be more common on the anterior and posterior lips of the cervix than at the lateral angles [11], [14], [15], [16], [17] while others reported a random distribution of CIN across the cervix [18]. If CIN does not arise randomly across the cervix, cervical biopsies could preferentially target the areas with highest CIN3 prevalence, improving CIN3 detection [18]. In our analysis, the largest study to date with LEEP endpoints to examine the distribution of cervical lesions, we showed that there was a uniform distribution of CIN3 LEEP segments across the cervix. Earlier studies relied only on colposcopically directed biopsy to study the topography of cervical lesions [19], [20]. The lack of randomness observed in these previous studies may not be related to the biology of CIN, but rather the unequal distribution of colposcopy guided biopsies and the mechanical ease of taking biopsies at 12 o'clock and 6 o'clock locations (25–26).

We also observed that compared to CIN3 cases with CIN2, solitary CIN3 and CIN3+CIN1 cases were more likely to have a larger sized CIN3 lesion. Our data corroborate the model that high-grade pre-cancer grows out from a small lesion possibly surrounded by low grade lesions, such that either CIN3 expands while the CIN1 regresses [21] or CIN3 expands and progressively replaces lower grade lesions [9]. Compared to other CIN3 subgroups, we observed that women without concomitant CIN2 or CIN1 lesions were more likely to be older and have longer sexual activity span, two highly correlated variables. These findings support that CIN3s have continuously spread circumferentially to different areas of the epithelium. The presence of CIN1 and CIN2 may indicate transient HPV infections that are more common among younger women. In support of this, we found that CIN3 without concomitant CIN2 or CIN1 were more likely to have fewer high risk HPV infections. This corroborates the clonal outgrowth of an HPV infection causing a lesion, while concurrent transient infections resolve spontaneously over time.

We sought to understand how clinical and pathological characteristics are related to CIN3 lesion size. The CIN3 lesions observed by McCredie and colleagues in their retrospective study of women with untreated CIN3 who progressed to cancer were large [5], in contrast to the small CIN3s observed in the intensively screened Atypical Squamous Cells of Undetermined Significance–Low-grade Squamous Intraepithelial Lesion (ASCUS-LSIL) Triage Study (ALTS) population [10]. Our analysis expanded upon these observations and examined the distribution of selected factors by CIN3 size in a complete referral population which included both women with LSIL and HSIL cytology results. Most importantly, we showed that fewer Pap screens in the past 5 years were more common among women with larger CIN3s, which suggests that less cytology screening and longer screening intervals allowed for longer undetected CIN3 growth. Unfortunately, we did not have information about the exact timing of the last cytology screen, which would have allowed estimating CIN3 growth rates. HPV16 has the highest carcinogenic potential and highest attribution to cervical cancers worldwide [22], [23]. Previous studies have suggested that HPV16-related CIN3 and cancer may be detected earlier than lesions related to other types [24], [25], [26]. It is unclear whether this is related to a faster growth of HPV16-related lesions or to a greater likelihood of HPV16-related lesions to cause cytologic abnormalities and abnormal colposcopic impression, facilitating detection in screening. Interestingly, in our population, HPV16 positivity was not associated with larger CIN3 lesion size, suggesting that although HPV16-related lesions may grow faster, they are detected as the same size as non-HPV16 lesions.

We did not observe associations between other risk factors previously reported to be associated with HPV infection and progression (number of sexual partners, OC use, parity, smoking) and CIN3 size, suggesting that these factors are not paramount at the later stages of CIN3 natural history. However, we noted an insignificant trend of less OC use in women with larger CIN3s. We recently observed in the same population that contraceptive methods requiring doctor visits such as OCs are associated with more Pap tests in the previous five years, which could explain this observation (data not shown).

In addition, we examined the effect of CIN3 size on cervical cancer screening results and found that larger CIN3 lesions were more likely to be diagnosed as HSIL+ at time of LEEP visit. Similarly, larger CIN3 size was more common among more severe colposcopic impression at time of LEEP visit and with a higher percentage of a CIN3+ biopsy result at the colposcopy visit, demonstrating that larger CIN3 cases are easier to detect by colposcopy. These finding highlight a current dilemma in cervical cancer screening: new screening tests such as HPV DNA detection are more sensitive than the current gold standard of following up cytology with colposcopy and biopsy [27]. Small high grade lesions may be picked up by HPV testing, but are missed at colposcopy.

The main strengths of our study are the large population-based sample of CIN3s and the detailed mapping of LEEP specimens, which allowed for a thorough evaluation of the heterogeneous manifestations of CIN3 cases. While examination of the 12 LEEP segments allowed us to study lesion size in unprecedented detail, a finer resolution would have provided more accuracy since multiple histologic diagnoses could be found even within a LEEP segment. Although the cross-sectional design of our study may be viewed as a limitation, it is not possible to follow CIN3 prospectively. Furthermore, this design permitted the accrual of large numbers of women into the study for studying CIN3 cases with detailed mapping of disease in LEEP specimens. In addition, our analysis was not based on panel adjudication of histology results, but on the community histology diagnosis by a single experienced pathologist.

In summary, our data showed that women with CIN3 lesions without concomitant CIN2 or CIN1 lesions were more likely to be older, have longer sexual activity span, and have fewer high risk HPV infections and that larger CIN3 lesions were more common among women infrequently screened, with HSIL or worse cytology, and CIN3 or worse impression in colposcopy. Interestingly, we also observed that in our population, HPV16 positivity was not associated with larger CIN3 lesion size. Although our and others' data suggest that CIN3 lesion size is an important indicator of risk of invasion, lesion size can only be determined post-treatment. We show that HSIL cytology and CIN3 impression in colposcopy with CIN3 biopsy results point to larger CIN3s that most likely have a higher risk of invasion compared to small incipient lesions. While the findings from this study are important, they are not sufficient to establish which CIN3 should be treated. We are now conducting detailed molecular analyses of cervical lesions, using microdissection to permit the molecular evaluation of CIN3 heterogeneity to identify better risk markers for management of CIN3.
